# Evaluation of truck driver rest locations and sleep quality

**DOI:** 10.5935/1984-0063.20210028

**Published:** 2022

**Authors:** Felipe Pereira Rocha, Elaine Cristina Marqueze, Göran Kecklund, Claudia Roberta de Castro Moreno

**Affiliations:** 1 University of São Paulo, Department of Health, Lifecycles and Society, School of Public Health - São Paulo - SP - Brazil.; 2 Catholic University of Santos, School of Public Health - Santos - SP - Brazil.; 3 University of Stockholm, Psychology Department, Stress Research Institute, Stockholm - Stockholm - Sweden.

**Keywords:** Sleep, Resting Places, Truck Drivers, Public Health

## Abstract

**Objectives:**

Truck drivers’ work organization requires that rest and sleep be taken in various locations, where sleep quality might be affected by the discomfort of these environments. The purpose of this study was to evaluate truck drivers’ rest locations and their association with sleep quality utilizing an ergonomic approach.

**Material and Methods:**

The sleep quality of 81 truck drivers was assessed using the Pittsburgh sleep quality index (PSQI). An adapted version of the ergonomics workplace analysis (EWA) instrument was used to evaluate 44 rest locations.

**Results:**

Half of the workers preferred sleeper berths (51.2%) as a rest place. Sleep was classified as poor by 71.6% of the drivers. Dorms were rated more positively (p<0.001) by truck drivers (2.0±1.1) than by the analyst (2.6±0.6). Sleeper berths and dorms were rated statistically different by truck drivers (p=0.002), as well as by the analyst (p=0.003). No correlation was found between EWA evaluations and total score for sleep quality. Separate analyses of dorms and truck berths showed very few correlations. The higher the noise of roommates in dorms, the worse the sleep quality. Conversely, noise in corridors or outside the room positively impacted sleep quality.

**Conclusion:**

Noise in the rest place may affect sleep in both directions, negatively or positively. Sleep was classified as poor regardless of resting place. The quality of resting places seemed to have little effect on sleep quality of truck drivers. Factors other than rest place, such as work scheduling, are probably more important for promoting good sleep quality.

## INTRODUCTION

Several studies have shown an increased risk of road accidents among long-haul drivers^1,2^; as well as high risk for morbidities such as diabetes^3^ and cardiovascular diseases^4^. In addition, truck drivers are exposed to high job demands and stressful features of the work environment, which may also contribute to an unhealthy lifestyle such as inadequate eating habits^5^ and high alcohol intake^6^. A common consequence of an unhealthy lifestyle, morbidities, and a strenuous work environment is poor sleep quality^4,7^.

Evidence suggests that truck drivers’ work situation promotes bad quality of sleep, excessive daytime sleepiness, and high prevalence of obstructive sleep apnea^8-10^. Moreover, some studies have suggested that transportation companies provide inadequate on-site rest areas, with unsafe places to rest, besides unhealthy food and bad toilet facilities^11-13^. Poor and unsafe rest areas may impair the quality of sleep which in turn may increase the occurrence of sleepiness behind the wheel.

Furthermore, the literature lack of recent evidences studying the relationship between quality of sleep and rest places. However, results of a study conducted at an Australian long-haul transportation company showed that sleep at dorms provides longer time in bed, higher sleep efficiency, less awakenings, and lower total wake time compared to truck berths^12^. Baulk and Fletcher (2012)^11^ observed longer sleep duration, better quality of sleep, and reduced fatigue levels when the truck driver was sleeping at home compared to sleeper berths.

Most studies evaluating rest locations have examined the quality of mattresses and bedding systems^14^ or the effect of environmental temperature on sleep^15^. However, there are several environmental factors that can affect the quality of sleep. In addition to thermal comfort, ambient noise and humidity are example factors that also need to be taken into account. In this context, an ergonomic approach used in the workplace could be an important means of assessing the quality of a rest location, as well as a step towards promoting better sleep and rest opportunities for long-haul truck drivers. An ergonomic analysis of the workplace typically includes a detailed quantitative and qualitative assessment of this environment. The evaluation is performed by a team involving researchers or health safety experts and workers^16^. This ergonomic approach provides unique information of the rest places by taking into consideration the evaluation and input of the truck drivers themselves, conferring reliability to the analysis, along with possible preventive suggestions.

Considering the aforementioned aspects, the aim of this study was to evaluate truck drivers’ rest locations (dorms and sleeper berths) and their association with sleep quality utilizing an ergonomic approach.

## MATERIAL AND METHODS

### Study design and participants

This is an ergonomic study conducted in a large Brazilian transportation company with branches in two different cities: São Paulo (population 12.2 million) and Campinas (population 1.2 million)^17^. The company is responsible for the distribution of goods in the computer industry, pharmaceutical industry, aviation and electronics, with a fleet of over 1,250 light, medium, and heavy vehicles.

Truck drivers filled out questionnaires collecting data on demographics (sex, age, and marital status), health (reported morbidities and body mass index), and lifestyle such as smoking and drinking. The Berlin questionnaire^18^ was used to estimate the occurrence of sleep apnea syndrome among the drivers. All participants were long-haul truck drivers that worked an irregular work schedule, including nights. This irregularity was due to large variability in workload and staffing demands. After driving all night, the drivers usually return to the company branches between 8:00h and 10:00h. After finishing administrative tasks, they go to bed at their rest place of preference, dorms or sleeper berths. The drivers usually return home after 10 to 15 days of work, which equates to two or three visits per month.

The sample size was calculated a priori using the G* Power software, version 3.1.9.2 (Kiel University, Germany). For the calculation, multiple linear regression, statistical significance of 5%, effect size of 0.15 (small effect), and sample power of 80% were considered. Thus, the sample size should be of 77 participants. A total of 127 truck drivers were invited to take part in the study, of which 81 agreed to participate. Most participants were interviewed at the São Paulo branch. Regarding the sample, all participants were male, mean age was 44 years (SD=10) and age range was 22-64 years.

The Pittsburgh sleep quality index (PSQI) was applied to assess truck drivers’ quality of sleep. This is a 19-item self-assessment tool of reported sleep behavior over the past month. Each composite score ranges from 0-3, with higher numbers suggestive of worse sleep quality in the respective domain. A total score >5 indicates poor quality of sleep. Therefore, our study results considered the PSQI ranges suggested by Buysse et al. (1988)^19^ and Bertolazzi et al. (2011)^20^. In this study, the PSQI was applied by asking the drivers to evaluate quality of sleep in general, which considers sleep in any situation^19,20^.

To assess the quality of truck drivers’ rest locations, an adapted version of the ergonomics workplace analysis (EWA) instrument was used. All participants and the researcher evaluated both truck berths and dorms, yielding a total of 81 evaluations, comprising 39 (48.1%) dorms and 42 (51.9%) truck berths. All rest place evaluations were performed separately by the analyst and the drivers.

The ergonomic evaluation considers the work task or workplace to be analyzed. The analyst rates the various factors on a scale, usually from 1 to 5. The value 1 is given when the situation has the smallest deviation from the optimal or generally acceptable condition. Values 4 and 5 indicate that the working condition or environment may eventually cause damage to workers’ health. In this study, a scale of 1 to 5 was also adapted and categorized in order to determine significant proportions: good (1), fair (2 and 3), and bad (4 and 5). The work task is usually divided into subtasks, which are analyzed separately. Separate analyses are required for each of the subtasks if they differ greatly. If the rating by the worker is very different from the researcher’s classification, the work situation should be analyzed in more detail^16^.

In the present study, the ergonomic evaluation was carried out by visiting each reported rest place (dorms or truck berths). The analyst inspected each rest place visually, noting information on room size, illumination, cushion, bed, mattress and sheets (dorms), sleeper berth size, illumination (truck), and type, year and model of truck. Considering the driver would evaluate the environment according to his perception, the analyst made a subjective analysis as well. This means that no device was used to evaluate noise, temperature, and illumination. Another important ergonomic step was to interview employees of different sectors in order to understand the dynamic of the transportation company and truck drivers’ work schedule. Observations were also important to allow a deeper understanding of the work dynamic. In addition, each truck driver’s evaluation was carried out during the analyst’s visit to the rest place locations, also utilizing the adapted EWA instrument as a script during the interview.

The school of public health ethics committee approved the study (number 2.995.488) and all participants involved signed a written consent to participate in the study.

### Data analyses

Descriptive statistics were reported as the mean and standard deviation for continuous variables and as frequencies and percentages for categorical variables. Student t-test, ANOVA, and Tukey post hoc test were performed to analyze differences between means. Spearman’s correlation coefficient was used to evaluate the correlation between PSQI score and EWA subjective evaluations followed by a multiple linear regression analysis (stepwise backward technique, only including variables with *p*<0.20) considering PSQI score as a dependent variable and analyst’s subjective score as an independent variable. In all tests, significance was considered when *p*<0.05. All data analyses were carried out using the software Stata, version 21.0 (StataCorp, Texas, U.S.) and SPSS, version 25.0 (SPSS Inc., Chicago, U.S.).

## RESULTS

### Demographic, work and health information

The prevalence of poor sleep quality among drivers was 71.6% and the mean sleep time was 6.3h (SD 1.9h). Comparison of demographic aspects according to sleep quality revealed statistically difference at age, which indicates that younger drivers are having poorer sleep quality compared with older ones (47 to 55 years) (Table 1).

**Table 1:** Sociodemographic, health, and work characteristics of truck drivers according to sleep quality score.

Variables Branches	PSQI means (SD)	ANOVA p-value
**Age range**		
27-39 years	8.3 (2.8)	0.05*
40-46 years	7.6 (2.7)	
47-55 years	6.0 (2.2)	
> 55 years	6.8 (3.2)	
**Years driving**		
1-7 years	8.0 (2.9)	0.12
8-15 years	6.4 (3.2)	
16-21 years	8.0 (2.6)	
> 22 years	6.7 (2.3)	
**BMI**		
Normal	7.9 (1.7)	0.80
Overweight	7.1 (2.8)	
Obese	7.4 (3.2)	
**City**		0.90^#^
São Paulo	7.2 (2.8)	
Campinas	7.3 (3.0)	
**Employment contract**		0.20^#^
Outsourced	7.5 (2.9)	
Formal	6.6 (2.6)	
**Shift**		0.50^#^
Day shift	8.3 (1.5)	
Night shift	7.3 (2.9)	
**Hours driving per day**		
≤8 hours	6.3 (3.1)	0.40
8-9.9 hours	7.4 (2.7)	
10-11.9 hours	7.5 (3.3)	
12-15.9 hours	8.7 (1.9)	
≥ 6 hours	7.0 (0)	

**Notes:**

* Tukey post hoc test *p*=0.046;

# Student t-test.

The Berlin questionnaire showed that 37% of drivers had suspected obstructive sleep apnea. However, there were significant associations of sleep apnea with sleep quality (x^2^=0.07, *p*=0.8) and with rest places (x^2^=1.267, *p*=0.26).

### Rest places

The truck drivers’ EWA evaluations were compared with the analyst’s evaluations. There were no differences (*p*=0.17) between average scores of all EWA aspects evaluated for sleeper berths by truck drivers (2.7±1.2) and by the analyst (3.1±0.8). On the other hand, the dorms were evaluated more positively (*p*<0.001) by truck drivers (2.0 ±1.1) than by the analyst (2.6±0.6). Sleeper berths and dorms were rated as statistically different by truck drivers (*p*=0.002), as well as by the analyst (*p*=0.003).

**Figure 1:**
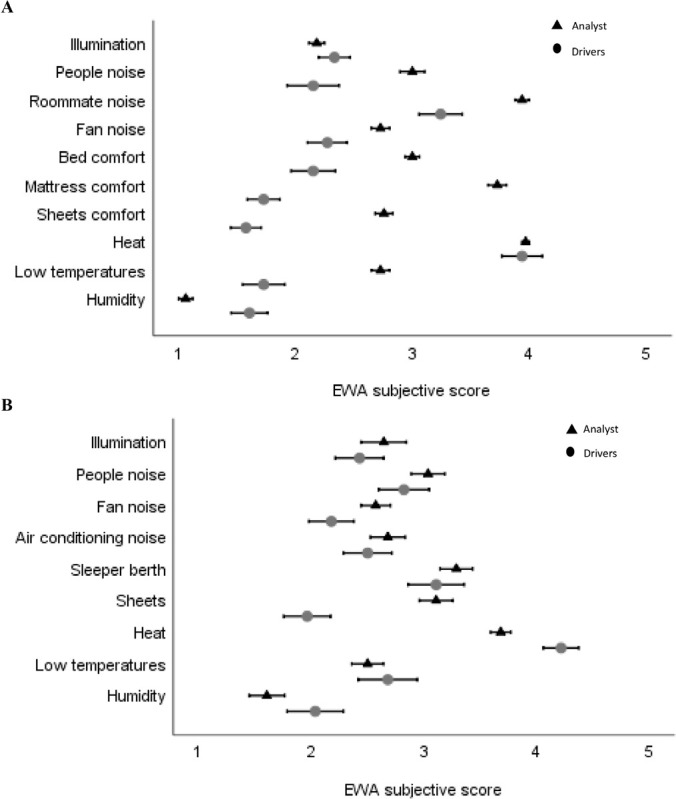
Analyst’s and truck drivers’ subjective evaluations according to EWA variable means (SE), for dorms (A) and truck berths (B).

The lowest score on the evaluations of sleeper berths was for heat (Figure 1B), and there was no difference between drivers and analysts. Regarding dorms, the lowest scores were obtained for heat, roommate noise, and mattress comfort (Figure 1A). For roommate noise and mattress comfort, the analyst reported a more negative score compared with the drivers.

**Figure 2:**
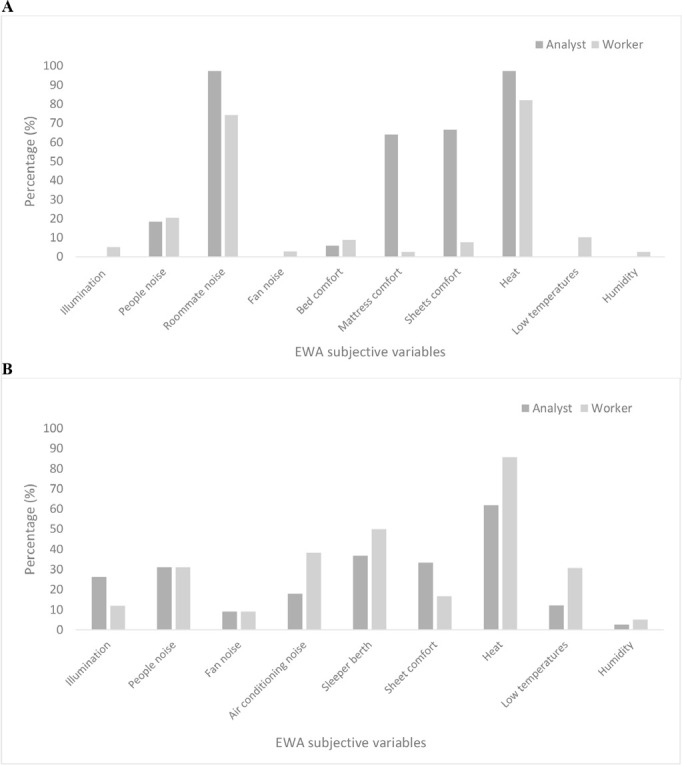
Analyst’s and truck drivers’ subjective evaluation proportions according to EWA variables with score >4 for dorms (A) and truck berths (B).

In general, the percentage of poorly rated dorms (score ≥4) by the analyst was higher compared to the drivers, which may explain the difference reported between their respective evaluations (Figure 2A). Truck berths seemed to be rated better by truck drivers (Figure 2B).

Just over half of the workers preferred trucks (51.2%) as a rest place. However, sleep was classified as poor by drivers for both places (dorms=6.8; truck berths=7.7), with no statistical difference between means (t=-1.432, *p*=0.47).

There were no statistically significant correlations between EWA and PSQI for dorms according to either the analyst’s or drivers’ evaluations (Table 2).

**Table 2:** Spearman’s correlation between PSQI score and EWA subjective evaluations for dorms only.

EWA variables	Analyst		Drivers	
	rho	p	rho	p
				
Illumination	0.15	0.36	-0.03	0.85
People noise	-0,25	0.13	0.06	0.70
Roommate noise	0.24	0.13	0.23	0.20
Fan noise	-0.03	0.85	-0.11	0.50
Bed comfort	0.09	0.59	-0.11	0.50
Mattress comfort	-0.08	0.61	-0.01	0.94
Sheets comfort	-0.06	0.73	-0.26	0.11
Heat	-0.09	0.60	0.00	0.98
Low temperatures	-0.08	0.61	0.08	0.64
Humidity	0.30	0.08	-0.03	0.86

A significant negative correlation was found between air conditioning noise and PSQI score according to the truck drivers’ evaluations; the noise produced by the air conditioning (in trucks) was associated with better sleep quality, where this was a borderline correlation (Table 3). No other significant correlations were found.

**Table 3:** Spearman’s correlation between PSQI score and EWA subjective evaluations for truck berths only.

EWA variables	Analyst		Drivers	
	rho	p	rho	p
Illumination	-0.09	0.56	0.12	0.44
People noise	-0.17	0.30	-0.28	0.07
Fan noise	0.19	0.24	-0.12	0.47
Air conditioning noise	-0.06	0.71	-0.32	0.05
Sleeper berth	-0.23	0.17	-0.05	0.74
Sheet comfort	-0.19	0.90	-0.03	0.84
Heat	-0.05	0.72	0.17	0.28
Low temperatures	-0.02	0.87	0.00	0.99
Humidity	-0.18	0.25	0.07	0.67

However, the linear regression showed that the noisier the roommates in dorms, the worse the quality of sleep. The same pattern was found for humidity, where the higher the humidity, the worse the sleep quality. Conversely, the same analysis showed a negative correlation between people noise and sleep quality. This indicates that noise in the corridor or outside the room impacted positively on sleep quality (Table 4).

**Table 4:** Multiple linear regression analysis* considering PSQI score as dependent variable and analyst’s subjective score as independent variable for dorms only.

Sleep quality	B	95% CI	p
People noise	-1.95	-3.74,-0.17	0.03
Roommate noise	4.16	0.80, 7.53	0.02
Humidity	3.18	0.22, 6.13	0.04

**Notes:**

* Adjusted for age and time working as truck driver; R=0.61; R^2^=0.37.

## DISCUSSION

This study aimed to evaluate truck drivers’ rest locations and their association with sleep quality utilizing an ergonomic approach. EWA data showed that, in general, dorms were evaluated more positively by truck drivers compared to the analyst, and truck berths seemed to be better evaluated by truck drivers. In spite of the interesting results regarding the EWA evaluations, we found no correlation between the ergonomic score and sleep quality ratings.

However, separate analyses of dorms and truck berths showed few significant correlations. First, there were no correlations between sleep quality and the dorm evaluations by both analyst and drivers. Regarding the sleeper berths in the trucks, only a borderline negative correlation between air conditioning noise and sleep quality evaluated by the drivers was found. This suggests that the noisier the truck air conditioning, the better the sleep. White noise devices have been described as having the potential for consolidating sleep in patients exposed to the intensive care unit in hospitals^21-23^. Also, it has been suggested that the individual’s ability to maintain sleep, while exposed to external noise varies according to the individual’s sleep spindle rate^24^. It is well-known that snoring disturbs the sleep quality of bedroom partners^25,26^.

The linear regression analysis also showed that the higher the noise of the roommates in dorms, the worse the sleep quality. Conversely, noise in the corridor or outside the room impacted positively on sleep quality. It seemed that noise may have affected sleep in both directions, i.e., negatively or positively, according to the individual, which would corroborate the study by Dang-Vu et al. (2010)^24^.

The linear regression model in this study revealed that the higher humidity in dorms, the worse the sleep quality. This result was expected since sleep environment variables such as high air temperatures, high relative humidity, air pollution, and CO_2_ concentrations may impair sleep, especially during the summertime^27,28^. In this context, hot and humid resting places may lead to increased stress levels, poor driving performance, and a stimulated central nervous system that in turn can contribute to chronic diseases^29,30^. Indeed, high temperatures are problematic to sleep especially when we consider that 96.3% of the drivers worked at night, meaning they were compelled to rest during the day. It is important to point out that data collection was performed during Brazil’s summertime, when temperatures are normally high.

To our knowledge, this is the first study that has used an ergonomic instrument to analyze rest places. Our findings show an agreement between the analyst and the drivers regarding the sleeper berth evaluations. In addition, both rated dorms different from sleeper berths. Nevertheless, there was a difference in relation to dorm evaluations, with better ratings given by the drivers than the analyst. The analyst was probably more rigorous in terms of general comfort evaluation than the drivers when both evaluated the dorms. This suggests that the dorms could be improved to reach a higher level of comfort.

Notwithstanding, the majority of truck drivers preferred the truck berths over dorms. This finding may be explained by the fact that drivers have to share rooms in the dorms, with no private rooms, and bedrooms are near restrooms or leisure rooms. People’s arrival at night was reported as a factor disturbing sleep, particularly due to door noise and illuminated corridors. Darwent et al. (2012)^12^ also observed a preference for sleeping in the truck berths among Australian truck drivers.

The prevalence of poor sleep quality was high among the drivers (71.6%) and their resting places (truck berths and dormitories) were rated poorly by both drivers and analyst. The prevalence of poor rating was high when compared to other studies^8,31,32^.

The present findings suggest that truck drivers’ rest places are important for their comfort and well-being during working hours, yet insufficient to obtain consistent good sleep quality. Kecklund and Åkerstedt (1997)^33^, for example, suggested that truck sleeper berths do not affect sleep negatively even in a noisy environment, suggesting that other variables should be further investigated. In this respect, it is likely that work organization factors negatively impact the sleep of truck drivers.

A recent study of American truck drivers suggests an association of sleep quality with organizational and behavioral factors, such as long working hours, shift work, smoking, sedentary, and high risk of developing cardiometabolic diseases^4,7^. Another important work organization factor was highlighted by Ulhôa et al. (2010)^34^ in Brazilian truck drivers. In the study, the presence of minor psychiatric disorders such as depression, anxiety, and fatigue were associated to work-related factors such as traffic congestion, tracking control, and extended working hours.

Furthermore, alcoholic beverage consumption and others illegal toxic substances may impact negatively at sleep behaviors among truck drivers as well as increased risk of traffic accidents^34-37^. For instance, the study from Leopoldo et al. (2015)^35^ showed that, from a sample of 684 Brazilian truck drivers, 67.3% reported alcohol use in the previous 30 days and 54.6% reported multiple drug consumption.

Indeed, at the work organization level, the gap between prescribed work and actual work can lead to physical, mental and psychological illness. Company policy must take into consideration the actual performed activity to devise individual or collective sickness prevention strategies. Thus, it is important to know and recognize the effects of work organization on worker health^35^. A deeper understanding of the variables in truck drivers’ rest places, together with organizational influences, is fundamental to achieve better work conditions. This knowledge can also promote effective more public policies that reflect, at least in part, the reality of the drivers and consequently help improve their quality of sleep.

It is important to point out some limitations of this study; the results should be regarded as a case study and therefore recommendations for other transportation companies should be made with caution. No control group was possible because all truck drivers had only two rest locations authorized by the transportation company and, consequently, no drivers slept only at home. As previously mentioned, sleep quality may be affected by numerous work organization factors, such as stress, schedule, lifestyle, etc. It is also important to point out that the PSQI classification was considered in general, since it was difficult for the drivers to rate sleep quality when they sleep in a specific place.

However, the results obtained by the present study have the potential to provide transportation companies with valuable information to devise better improvement strategies for the evaluation of rest places. Additionally, truck drivers work organization must be considered to attenuate the gap between prescribed and actual work, thereby contributing to worker well-being.

## CONCLUSION

In summary, this is the first study to use an ergonomic instrument to evaluate the quality of truck drivers’ rest places. The majority of the drivers reported poor sleep quality regardless of resting places. Noise may affect sleep quality in both directions, negatively or positively, according to the individual. The quality of resting places seemed to have a low effect on the sleep quality of the truck drivers. It is likely that work scheduling and work environment characteristics are more important factors for truck drivers’ sleep quality.
